# Antimicrobial Photoinactivation of In Situ Oral Biofilms by Visible Light Plus Water-Filtered Infrared A and Tetrahydroporphyrin-tetratosylate (THPTS)

**DOI:** 10.3390/microorganisms9010145

**Published:** 2021-01-11

**Authors:** Lamprini Karygianni, Sandra Ruf, Elmar Hellwig, Marie Follo, Kirstin Vach, Ali Al-Ahmad

**Affiliations:** 1 Clinic of Conservative and Preventive Dentistry, Center of Dental Medicine University of Zurich, CH-8032 Zürich, Switzerland; Lamprini.Karygianni@zzm.uzh.ch; 2 Department of Operative Dentistry and Periodontology, Medical Center, Faculty of Medicine, University of Freiburg, 79106 Freiburg, Germany; sandraruf@t-online.de (S.R.); elmar.hellwig@uniklinik-freiburg.de (E.H.); 3 Lighthouse Core Facility, Department of Hematology, Oncology & Stem Cell Transplantation, Faculty of Medicine, Medical Center, University of Freiburg, 79106 Freiburg, Germany; marie.follo@uniklinik-freiburg.de; 4 Institute for Medical Biometry and Statistics, Faculty of Medicine and Medical Center, University of Freiburg, 79104 Freiburg, Germany; kv@imbi.uni-freiburg.de

**Keywords:** antimicrobial photodynamic therapy (aPDT), water-filtered infrared A (wIRA), tetrahydroporphyrin-tetratosylate (THPTS), oral biofilm

## Abstract

The aim of this study was to examine the effect of aPDT with visual light (VIS) + water-filtered infrared A (wIRA) as a light source, and tetrahydroporphyrin-tetratosylate (THPTS) as a photosensitizer on in situ initial and mature oral biofilms. The samples were incubated, ex situ, with THPTS for two minutes, followed by irradiation with 200 mW cm − 2 VIS + wIRA for five minutes at 37 °C. The adherent microorganisms were quantified, and the biofilm samples were visualized using live/dead staining and confocal laser scanning microscopy (CLSM). The THPTS-mediated aPDT resulted in significant decreases in both the initially adherent microorganisms and the microorganisms in the mature oral biofilms, in comparison to the untreated control samples (>99.99% each; *p* = 0.018 and *p* = 0.0066, respectively). The remaining vital bacteria significantly decreased in the aPDT-treated biofilms during initial adhesion (vitality rate 9.4% vs. 71.2% untreated control, 17.28% CHX). Of the mature biofilms, 25.67% remained vital after aPDT treatment (81.97% untreated control, 16.44% CHX). High permeability of THPTS into deep layers could be shown. The present results indicate that the microbial reduction in oral initial and mature oral biofilms resulting from aPDT with VIS + wIRA in combination with THPTS has significant potential for the treatment of oral biofilm-associated diseases.

## 1. Introduction

Photodynamic therapy (PDT), a method which involves combining non-toxic, photosensitizing dyes with a light source, is becoming increasingly more important due to the emergence of multi-drug resistance in pathogenic bacteria, and due to the enhanced resistance of bacteria located in biofilms. Periodontitis, peri-implantitis and caries are the most common biofilm-associated infections in the field of dentistry. As has previously been reported, biofilm bacteria have an enhanced antibiotic resistance of up to 1000-fold that of planktonic bacteria [1]. Antimicrobial PDT (aPDT) as an alternative approach or adjunct method of dental bacterial decontamination, has the advantage that it can be applied locally because the photosensitizer is favorably located in the bacteria and not in the surrounding tissue [2]. Moreover, this therapy does not induce any resistance [1]. The mechanism of action in aPDT is, put briefly, that the presence of a photosensitizer, oxygen, and light induces oxidative damage in bacterial cells by generating free radicals or singlet oxygen [3,4]. Our previous studies examined photodynamic antimicrobial inactivation using toluidine blue, chlorine e6, indocyanine green and hypericin, respectively, as photosensitizers, in combination with water-filtered infrared A (wIRA) as a light source. These experiments showed that aPDT with wIRA has a remarkable potential for eradicating both initial and mature biofilms [1,5,6,7,8]. In addition, it altered the microbial composition within the biofilms. In contrast to other light sources used for aPDT, wIRA has the advantages of a higher level of perfusion, lower thermal stress, and eeper tissue penetration, promoting wound healing and protecting the external tissue layers [9,10].

In the present study, we evaluated the photodynamic efficacy of tetrahydroporphyrin-tetratosylate (THPTS) on initial oral bacterial adhesion and mature oral biofilm, by using visible light plus wIRA (VIS+wIRA) as the light source. THPTS is a tetracationic charged and water-soluble tetrahydroporhyrin dye, which has strong absorption in the infrared region (760.5 nm) [11]. In addition, this positively charged dye is taken up in large quantities by negatively charged mitochondrial membranes, which plays a major role in apoptosis by activating the caspase cascade [2,12]. Previous results have demonstrated that THPTS has a bactericidal effect on Gram-positive, methicillin-sensitive and methicillin-resistant *Staphylococcus aureus*, as well as on Gram-negative bacteria [2,13].

To date, no reported studies have examined the survival of microorganisms within initial and mature oral biofilms after the application of aPDT using VIS+wIRA as a light source and THPTS as a photosensitizer. The aim of this study is to examine whether aPDT with VIS+wIRA and THPTS causes any changes in colonization, biofilm formation and cell vitality, and to learn whether this method could improve the treatment of biofilm-associated oral infections in the future.

## 2. Materials and Methods

### 2.1. Study Participants and Test Samples

Six healthy volunteers aged between 25–54 years, gave written informed consent and were allowed to participate in the study. The Ethics Committee of the University of Freiburg (Nr. 91/13) reviewed and approved the study protocol. A clinical oral examination was conducted prior to the assays. Lactate formation rates of 2.5 ± 0.6 (scale from 1 to 9) were detected and saliva flow rates were measured at 1.2 ± 0.3 mL/min, whereas DMFT values (decayed, missing, filled teeth) were estimated at 4.5 ± 3. The grounds for exclusion from the study were: (1) systemic antibiotic use or use of antimicrobial mouth rinses e.g., chlorhexidine (CHX), during the last month before the study, (2) active carious lesions or periodontal disease, (3) pregnancy or lactation, (4) salivary gland disease, or (5) other severe, systemic disease.

To prepare the test samples, the buccal surfaces of bovine incisors from freshly slaughtered 2-year-old cattle were detached and modified into cylindrical enamel samples (diameter 5 mm, 19.63 mm^2^ surface area, height 1 mm) [8]. Examination of the cattle with the IDEXX laboratories BSE diagnostic kit (Ludwigsburg, Germany) prior to tooth extraction, excluded the presence of bovine spongiform encephalopathy (BSE). A wet grinding machine (Knuth-Rotor-3, Streuers, Willich, Germany) was then used to polish the sample enamel surfaces with sandpaper (abrasive grading scales from 250 to 4000 grit) in decreasing order of grain size. The polished bovine enamel slabs (BES) were checked under a light microscope (Wild M3Z, Leica GmbH, Wetzlar, Germany) and, finally, disinfected. The BES disinfection protocol included ultrasonication in NaOCl (3%) for 3 min to remove the superficial smear layer, air drying and then ultrasonication in 70% ethanol for 3 min. The BES were then ultrasonicated twice in double distilled water for 10 min and finally, placed in distilled water for 24 h to hydrate before use [1].

**Figure 1:**
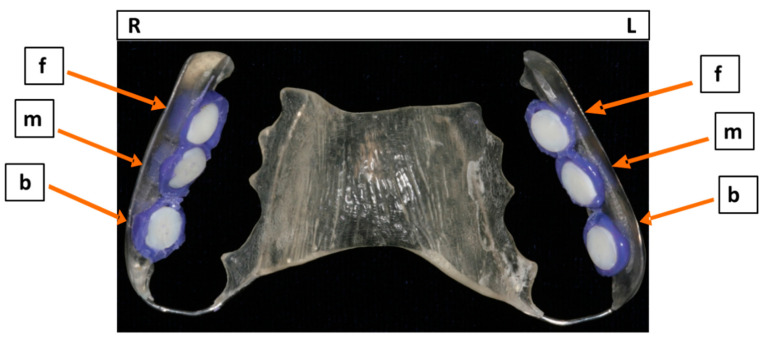
Upper jaw acrylic appliance with six bovine enamels slabs (BES) fixed with silicone to individually designed slots situated on both sides, right (R) and left (L) of the appliance. The enamel surfaces of the BES were placed in different locations at the front (f), in the middle (m) and at the back (b) of the appliance.

Individual upper jaw acrylic appliances were prepared for each study participant, and six BES were fixed on their approximal sites with an A-silicon compound (Panasil^®^ initial contact X-Light, Kettenbach GmbH & Co. KG, Eschenburg, Germany). The BES surfaces were exposed to the oral cavity after their margins had been completely covered by the impression material (Figure 1). The BES were then attached to the interdental area between the upper premolars and molars, in order to avoid disturbing the biofilm by movements of the tongue or cheek. Each study participant wore the acrylic appliances incorporating the BES twice, for 2 h or 3 days, respectively, and therefore carried a total of twelve BES. Initial and mature oral biofilm samples were formed, in vivo, in each volunteer’s oral cavity. The biofilm samples were then treated using the aPDT, ex vivo. 

### 2.2. Photosensitizers and Light Source

A broad-band VIS + wIRA radiator (Hydrosun 750 FS, Hydrosun Medizintechnik GmbH, Müllheim, Germany) with a 7 mm water cuvette was applied and an accessory orange filter, BTE31, was adjusted to the light generator. In general, the fitting of the BTE 31 filter allowed more than a double weighted effective integral irradiance, in terms of protoporphyrin IX’s absorption spectrum, in contrast to a standard BTE 595 orange filter. In particular, the continuous water-filtered spectrum covered a wavelength range from 570 nm to 1400 nm, with local minima of 970 nm, 1200 nm and 1430 nm, respectively, as a result of absorption by the water molecules [14]. The unweighted (absolute) irradiance of 200 mW cm^−2^ VIS + wIRA consisting of approximately 48 mW cm^−2^ VIS and 152 mW cm^−2^ wIRA, was applied to the samples for 5 min.

This broadband light source facilitated optimal light absorption by the photosensitizer used, which was tetrahydroporphyrin-tetratosylate (THPTS) (C_72_H_70_N_8_O_12_S_4_, TetraPDT Inc., Rackwitz, Germany). The photosensitizer THPTS was diluted in 0.9% saline solution (NaCl), to a final concentration of 100 µg ml^−1^. To prevent any light-induced photochemical alterations, the freshly prepared THPTS solution was stored in the dark at 4 °C for a maximum of 14 days. THPTS absorbs with an extinction coefficient of e= 105,000 M^−1^cm^−1^ at 760.5 nm in water and is considered to be a chemically stable, water soluble, highly pure (99.9% HPLC) and positively charged compound [2].

### 2.3. APDT Protocol for Oral Biofilms

**Figure 2:**
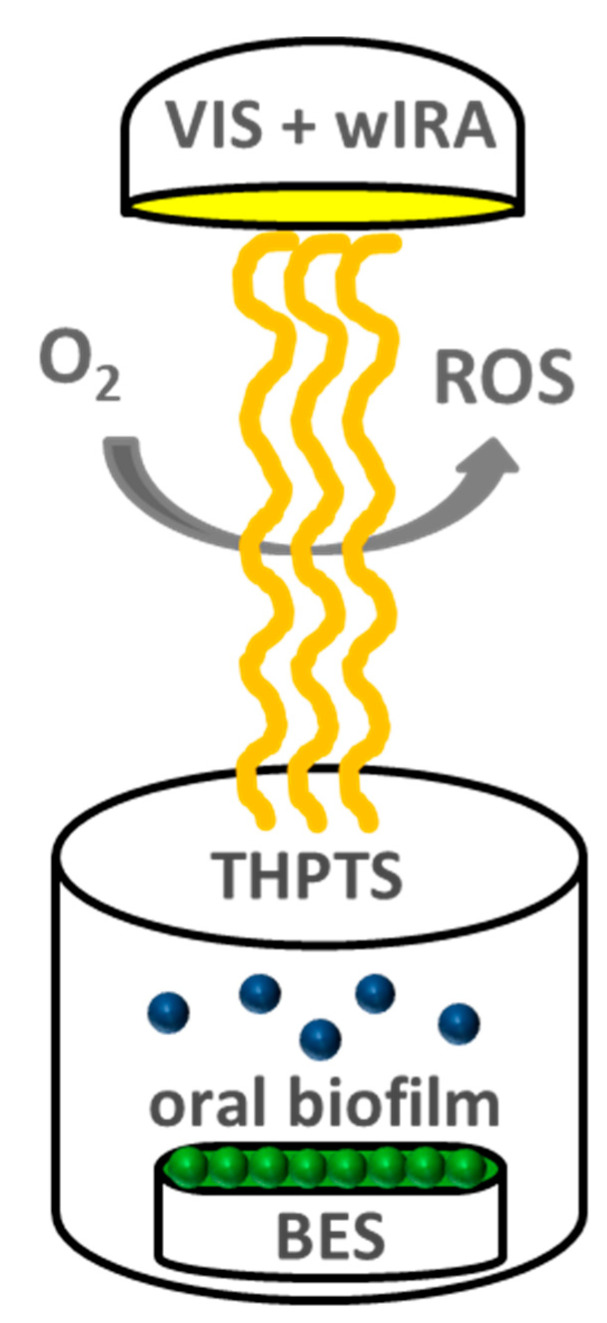
Simplified illustration of the antimicrobial photodynamic therapy (APDT) using visible light plus water-filtered infrared-A (VIS+wIRA). The photosensitizer tested, tetrahydroporphyrin-tetratosylate (THPTS), went to the excited-singlet state after being treated with VIS+wIRA at a wavelength range from 570 nm to 1400 nm. In the presence of oxygen (O2), the excited THPTS stimulated the release of reactive oxygen species (ROS), such as singlet oxygen, which penetrate and eradicate resistant microbial networks such as the oral biofilm.

Each study participant wore an individual, upper jaw, acrylic appliance with six BES for 2 h or 3 days, respectively. This procedure was performed twice for each volunteer and time period. Sterile tweezers were then used to detach the BES, with their in situ oral biofilms, from the acrylic appliances, and the BES were rinsed with sterile 0.9% NaCl for 30 s. Four specimens from the total of six BES per volunteer, served as controls. Specifically, two untreated biofilm samples served as negative controls and two 0.2% CHX-treated biofilm samples were used as positive controls. The remaining two biofilm-covered BES were treated with VIS + wIRA in the presence of 100 µg ml−1 THPTS. The BES were transferred into multiwell plates for the aPDT (24-well plate, Greiner bio-one GmbH, Frickenhausen, Germany) and incubated with THPTS for 2 min in the dark, in duplicate, with VIS + wIRA radiation being applied for 5 min at 37 °C (Figure 2). Thereafter, the BES were placed into multiwell plates with 1 mL 0.9% NaCl and the adherent microorganisms were quantified by determining the colony forming units (CFU). Additional biofilm samples were collected from a second cycle for each time point and visualized using live/dead staining. 

### 2.4. Quantification of Adherent Oral Biofilm Microorganisms

Small, sterile, foam pellets (Voco GmbH, Cuxhaven, Germany) were used to brush off the reverse dentine surfaces of the BES and their upright side margins. The BES were washed with 1 mL 0.9% NaCl for 10 s to remove non-adherent microorganisms, and then inserted into sterile Eppendorf tubes (Eppendorf GmbH, Wesseling-Berzdorf, Germany) with 1 mL 0.9% NaCl, ultrasonicated for 2 min in 1 mL NaCl on ice, and finally vortexed for 30–45 s. The suspensions of untreated BES (negative control) and CHX-treated BES (positive control) were then serially diluted up to 1:10^3^ in 0.9% NaCl. An equivalent dilution series (10^−1^ to 10^−3^) was also used for the aPDT-treated BES. Columbia blood agar plates (CBA, Becton Dickinson, Heidelberg, Germany) were then used to cultivate aerobic and facultative anaerobic bacteria at 37 °C and 5–10% CO_2_ for 5 days. Anaerobic bacteria were plated on yeast-cysteine blood agar plates (HCB, Becton Dickinson, Heidelberg, Germany) at 37 °C for 10 days (anaerobic chamber, Genbox BioMérieux SA, Marcy/Etoile, France). The number of colony-forming units (CFUs) per ml was determined using the Gel Doc EQ Universal Hood (Bio-Rad Life Science Group, Hercules, USA). Each measurement was repeated twice. 

### 2.5. Live/Dead Staining and Confocal Laser Scanning Microscopy (CLSM)

Fluorescent SYTO^®^ 9 stain was used with propidium iodide (PI) (Live/Dead^®^ BacLight™ Bacterial Viability Kit, Life Technologies GmbH, Darmstadt, Germany) to determine cell viability [15]. The green fluorescence stain, SYTO^®^ 9, can penetrate both intact and disrupted cell membranes whereas disrupted cell membranes are selectively permeable by red-fluorescent PI. As a result, viable bacterial cells fluoresce green and non-intact cells fluoresce red. Briefly, the SYTO^®^ 9 and PI were diluted in 0.9% NaCl to a final concentration of 0.1 nmol mL^− 1^. The aPDT-treated BES were then transferred to multiwell plates and stained with 1 mL SYTO 9/PI solution in 0.9% NaCl per well, in a dark chamber, for 10 min at room temperature. The stained BES were then placed, face down, onto a drop of NaCl solution in a chambered cover glass (µ Slide 8 well, ibidi GmbH, Munich, Germany) and analyzed using confocal laser scanning microscopy (CLSM, Leica TCS SP2 AOBS, Mannheim, Germany) with a 63 × water immersion objective (HCX PL APO/bd. BL 63.0 × 1.2 W, Leica, Mannheim, Germany). To quantify the biofilm vitality upon aPDT, the initial (2 h) and mature (3 day) biofilms were screened at three representative positions. Three positions for each of the 6 BES per subject, and therefore, a total of 18 biofilm locations, were screened for each time period. The upper and lower boundaries of the oral biofilm at each of the three selected locations were determined to measure the mean biofilm thickness. Thereafter, the aPDT-treated biofilms were scanned in the Z-direction at these three points, yielding optical-sections with a thickness of approximately 0.5 µm, each taken at 2-µm intervals throughout the biofilm layers. Sequential scanning was used to minimize the risk of spectral overlap. Each standard image was transformed into a digital image with a resolution of 1024 × 1024 pixels. The zoom setting was 1.0 which corresponds to physical dimensions of 140 × 140 µm. The measurements were conducted in duplicate. 

### 2.6. Image Analysis

The image analysis of the initial adhesion samples was conducted with the aid of the program Zen (Zeiss, Oberkochen, Germany) to yield maximal projections for each image stack, in order to quantify the covering grades of the scanned points. The Z-stacks for the 3 day biofilms were split into separate red and green stacks, of which each Z-layer was analyzed [1]. The image analysis program MetaMorph 6.3r7 (Molecular Devices Corporation, Sunnyvale, USA) was used for both the initial adherent and 3 day biofilms, to define the total surface area colonized by viable and non-viable microorganisms by manually setting intensity thresholds for each channel for each of the biofilm regions Measured. The covering grades of live and dead cells (% positive within total scanned biofilm region) were further analyzed.

### 2.7. Statistical Analysis

The means and standard deviations were computed for a descriptive evaluation of the data. The Friedman test was applied to evaluate the overall microbial load among the test groups. A *t*-test with Bonferroni correction (multiple testing) was used for pair-wise group comparisons, mainly because of a non-parametric test’s limited power in relation to small sample size. Diagrams of the viable bacterial counts on the log_10_ scale per square centimeter (log10/cm2) were visualized, stratified according to biofilm age (initial/mature). An analysis of variance (ANOVA) was conducted to analyze the differences between the vitality results for the aPDT-treated biofilms and the controls. The P-values were adjusted using Scheffé’s method. For each test group (control, CHX, THPTS), the continuous response variable was demonstrated as a boxplot of the viable oral microorganisms and separately from biofilm age (initial/mature). All the calculations were performed with the statistical software STATA 13.1.3. 

## 3. Results

### 3.1. Initially Adherent Microorganisms Decreased Significantly after aPDT

**Figure 3:**
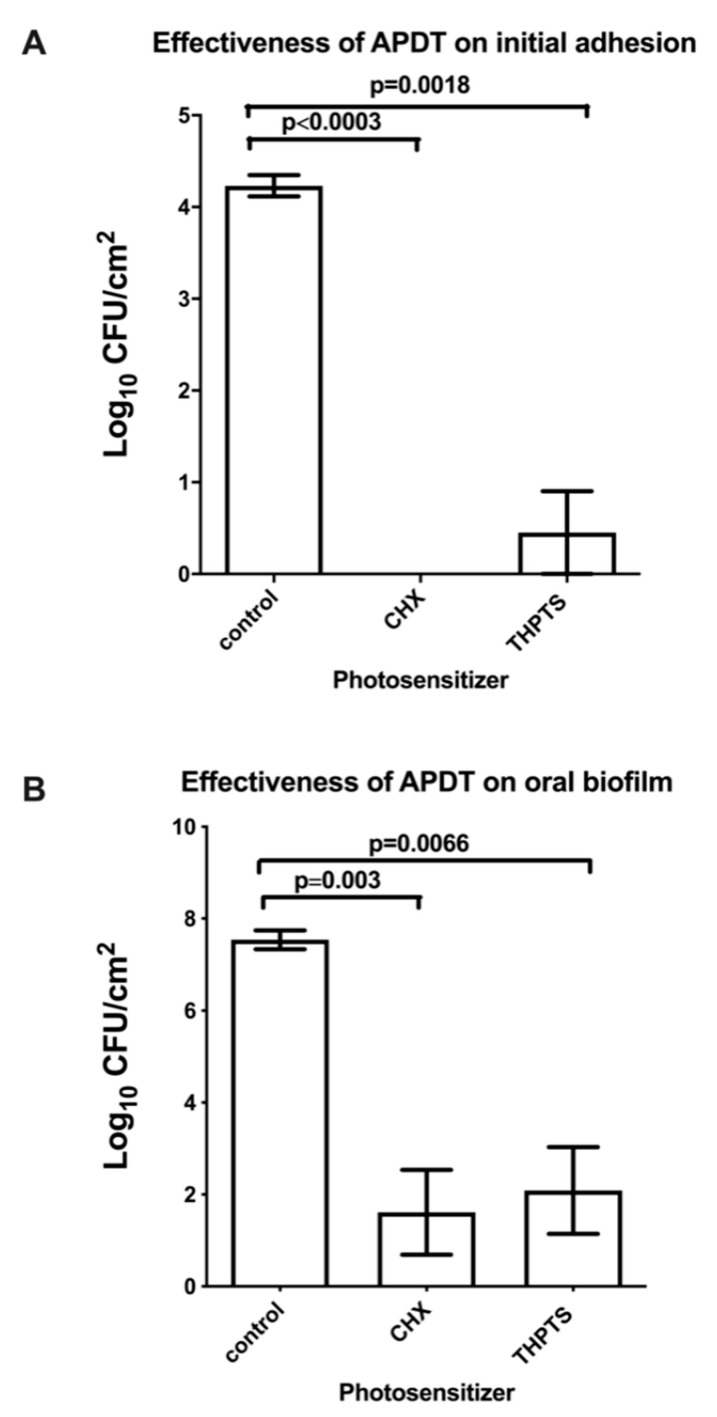
Columns depicting the colony forming units (CFUs) of oral bacteria after the antimicrobial photodynamic therapy (aPDT), during a two-hour initial adhesion (**A**) and three-day biofilm formation (**B**), respectively. Tetrahydroporphyrin-tetratosylate (THPTS) served as a photosensitizer. Untreated and 0.2% chlorhexidine-treated (CHX) samples were also tested as negative and positive controls, respectively. The CFUs are shown on a log10 scale per square centimeter (Log10/cm^2^). The significant *p*-values (*t*-test) are noted on the graphs.

Figure 3A shows the high eradication rates for initially adherent oral microorganisms after aPDT using VIS + wIRA in the presence of THPTS, plus the untreated negative and positive (CHX) controls. The THPTS-mediated aPDT resulted in a significant decrease, of more than 99.99%, in the viable microbial counts after two hours. In particular, the untreated control revealed a log10 CFU value (Log10) of 4.23 ± 0.28 (median 4.25), whereas no microorganisms survived in the CHX-treated biofilms. The aPDT with THPTS yielded a significant reduction (*p* = 0.0018) of 3.78 CFU (mean 0.45 ± 1.10,) on a log10 scale.

### 3.2. Viable Cells in Mature Oral Biofilms Significantly Decreased after aPDT

Figure 3B demonstrates the elevated elimination rates for microorganisms in the mature (three day) oral biofilm after aPDT treatment with VIS + wIRA in the presence of THPTS, as well as those of the untreated negative and positive (CHX) controls. The viable cells in oral biofilms decreased significantly after aPDT combined with THPTS, reaching a minimum reduction of 99.9% in the three-day-old biofilm. In comparison with the untreated negative control, which yielded CFU values (Log10) of 7.53 ± 0.50 (median 7.49), aPDT with THPTS (mean 2.08 ± 2.30) showed a significant reduction (*p* = 0.0066) in CFU counts of 5.45 log10. No cultivable anaerobic bacteria were detected after treatment with CHX (positive control). The three-day CHX-treated biofilms (mean 1.61 ± 2.25) showed a reduction in CFU (*p* > 0.1) comparable to that seen in the aPDT-treated groups.

### 3.3. THPTS-Mediated aPDT Interfered Significantly with Cell Vitality in Oral Biofilms

**Figure 4:**
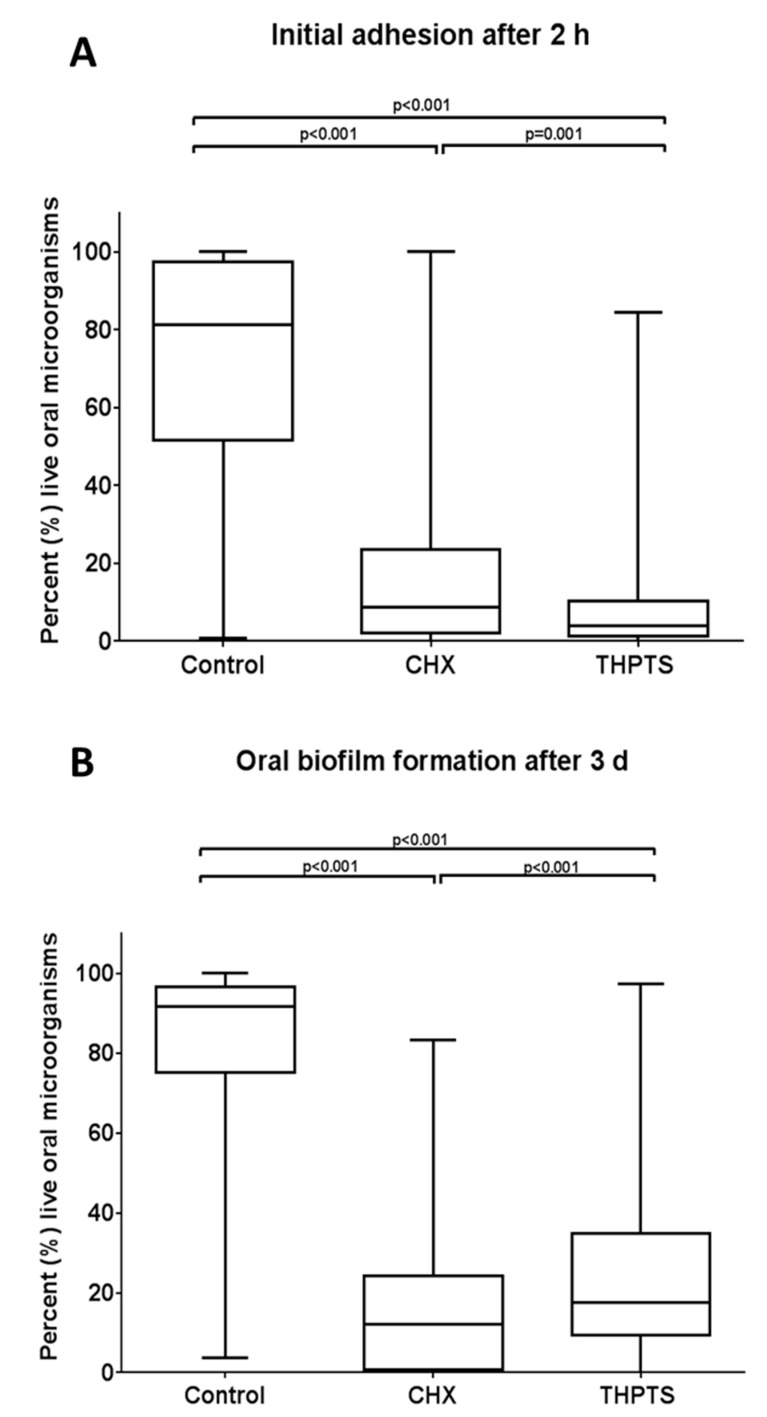
Boxplots demonstrating vitality percentages of oral bacteria during initial adhesion (**A**) and biofilm formation (**B**), respectively, as revealed by live-dead staining after the antimicrobial photodynamic therapy (aPDT). Tetrahydroporphyrin-tetratosylate (THPTS) served as a photosensitizer. Untreated and 0.2% chlorhexidine-treated (CHX) samples were also tested as negative and positive controls, respectively. The central line shows the median; whiskers indicate the minimum and maximum. The data’s significant *p*-values (ANOVA, Scheffé adjustment) are noted on the graphs.

The quantitative results for the remaining vital bacteria detected by the live/dead assay during initial adhesion (2 h) and oral biofilm formation (3 d) after APDT using VIS + wIRA and THPTS, are shown in Figure 4, in the form of boxplots. 

During initial adhesion, 71.20% of the untreated BES (negative control) was covered with viable microorganisms (Figure 4A). The statistical analysis revealed a significant decrease (*p* < 0.001) in the vitality rates when comparing the aPDT-treated biofilms (9.40%) and the untreated control. Interestingly, significantly more bacteria (17.28%) remained vital after treatment with CHX than in the aPDT-treated group. 

The use of aPDT using VIS + wIRA and THPTS in mature biofilms showed significantly decreased vitality rates (*p* < 0.001) of 25.67%, in comparison to the untreated biofilms (81.97%) (Figure 4B). However, significantly fewer microorganisms (16.44%) remained vital after treatment with CHX, compared to the aPDT-treated group.

### 3.4. CLSM Images Revealed High Permeability of THPTS within aPDT-Treated Oral Biofilms

**Figure 5:**
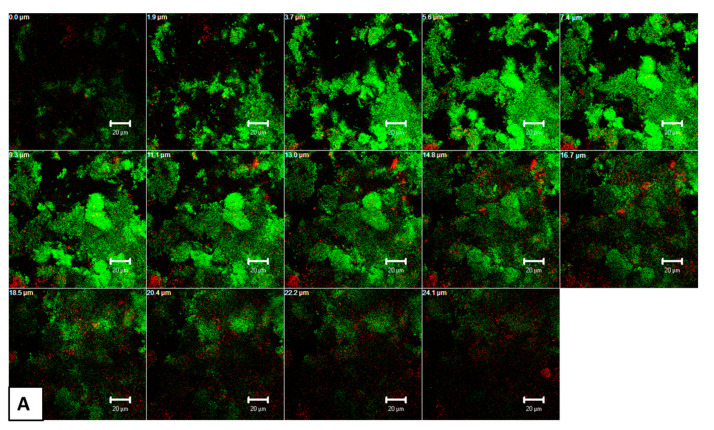

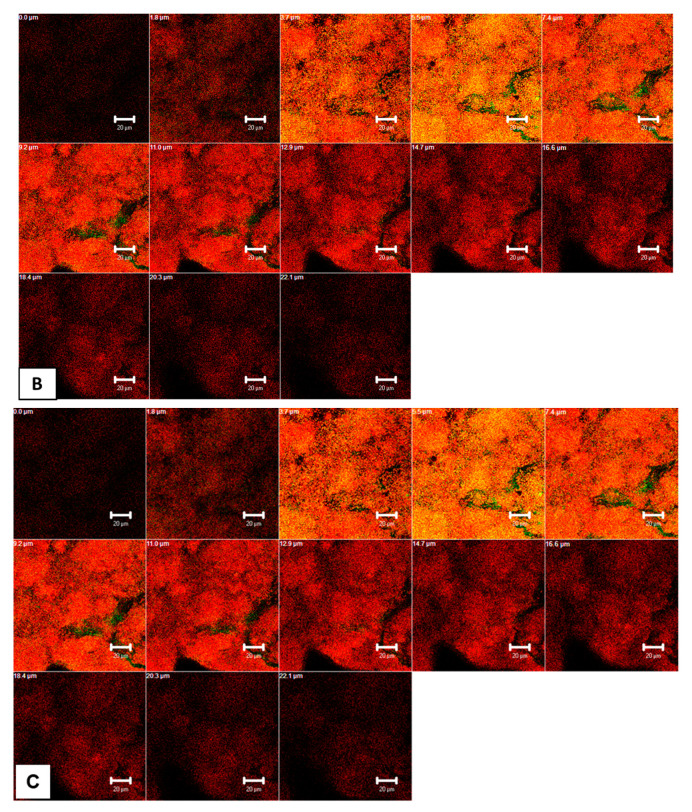
Representative confocal laser scanning microscopic (CLSM) images after live/dead staining, demonstrate the application of antimicrobial photodynamic therapy (aPDT) on oral biofilm formation (3 d). The live (green) and dead (red) oral bacteria are depicted on the Z-section panels of the untreated negative control (**A**), 0.2% chlorhexidine-treated (CHX) positive control (**B**) and the biofilms treated with aPDT in the presence of tetrahydroporphyrin-tetratosylate (THPTS) (**C**). Multiple Z-sections were created by vertical sectioning in 2 μm intervals through the biofilm above the enamel surface, as shown in panels (**A**–**C**). Scale bar for all images is 20 µm.

Figure 5 shows representative, cross-sectional CLSM images of mature oral biofilms (3 d) after live/dead staining, following aPDT with VIS + wIRA and THPTS. The untreated biofilms showed several, densely situated viable cells (green) (Figure 5A) in different configurations, while the few non-viable cells (red) related to homeostasis. Nevertheless, the positive controls contained mainly red-stained cells after treatment with 0.2% CHX, indicating a significant decrease in cell vitality (Figure 5B). The green-stained areas within CHX-treated biofilms imply the presence of persistent microbial cells. Similarly, the biofilms treated with aPDT and THPTS (Figure 5C) also contained numerous non-viable (red) cells, with no detectable differences in the biofilm thickness compared to that in the CHX-treated group. Interestingly, the Z-section CLSM galleries revealed the high permeability of THPTS following aPDT, even extending into the deep layers of the biofilm.

## 4. Discussion

In the present study, it was possible to demonstrate by live/dead staining, determination of colony forming units and confocal laser scanning microscopy, that aPDT with VIS + wIRA as the light source and THPTS as the photosensitizer, effectively reduces the microorganisms in initial and mature biofilms formed in situ. The effectiveness of VIS + wIRA in combination with other photosensitizers, such as indocyanine green, Hypericum perforatum extract, toluidine blue and chlorine e6, have already been examined extensively regarding oral biofilms [1,5,6,7,8]. The results of these studies have shown that aPDT using VIS + wIRA has a remarkable potential for eradicating both initial and mature biofilms and indicates that a combination of this light source and appropriate photosensitizers, seems to be an effective alternative or supplemental method of treating oral biofilm-associated infections such as caries, periodontitis or peri-implantitis. The advantages of VIS + wIRA over other light sources (e.g., LED, wide-band halogen lamps) as part of the aPDT have been reported [5,16]. Briefly, while LEDs have restricted emission wavelength spectra, and wide-band halogen lamps can induce tissue overheating, VIS + wIRA with an accessory orange filter, has a wavelength spectrum of between 570–1400 nm, protects external tissues by decreasing thermal stress due to significant subcutaneous tissue penetration, while the oxygen partial pressure in tissues is increased, leading to a higher perfusion rate, better wound healing, and pain reduction [9,10,17]. In particular, the use of LEDs in the treatment of oral diseases requires a long irradiation time which could lead to damage to the mucosal membranes and tissue within the oral cavity. 

THPTS, a cationic, water-soluble photosensitizer was investigated in this aPDT study because it has a strong absorption band in the infrared region (760 nm). A further advantage of this photosensitizer is that due to its cationic properties, it exhibits a high accumulation in negatively charged mitochondrial membranes, which play a key role in apoptosis [2,12,18]. In addition, THPTS shows deep tissue penetration (up to 15 mm) and is rapidly removed from healthy cells, reducing the skin’s photosensitivity and the energy required for photoactivation. Consequently, the potential tissue damage is decreased [11,19]. To date, there are very few studies which use THPTS in the field of aPDT but none with VIS + wIRA as a light source. Berndt-Paetz et al. [19,20] and Walther et al. [21], for example, performed the aPDT using a diode laser to examine the influence of THPTS on malignant neoplasia. The results showed that THPTS in human bladder carcinoma cell lines or rat urothelial carcinoma cells induces growth arrest, apoptosis and necrosis, and also has a cytotoxic effect on retinoblastoma cells, respectively. Diode lasers have also been investigated in aPDT with THPTS in the context of examining photodynamic efficiency against Gram-positive *Staphylococcus aureus* strains (MSSA and MRSA) and Gram-negative strains of *Escherichia coli* and *Pseudomonas aeruginosa* in bacterial suspension, resulting in a ≥ 99.999% decrease in viable bacteria [2]. In our study, the irradiation with VIS+wIRA after THPTS incubation, resulted in a comparable reduction of viable cells, in both initial (>99.99%) and mature (>99.9%) oral biofilms, although the incubation time and concentration of THPTS was lower in our study (2 min vs. >30 min, 100 µg/mL vs. 137µg/mL). Taking into account that the microorganisms in the biofilm are less accessible to substances than are bacteria in suspensions, this high percentage reduction shows that THPTS penetrates deep tissue, as well as having a high potential for photoinactivation. THPTS’s deep tissue penetration and the microbial eradication due to photoinactivation, could also be confirmed by confocal laser scanning microscopy after live/dead staining. Numerous non-viable cells were visible in the mature biofilms treated with aPDT and THPTS, even in deep layers of the biofilm. However, it should be considered that the light used would not be able to reach all the deeper layers of the biofilm, leading to a weaker antimicrobial effect against biofilm microorganisms in comparison to CHX.

The quantification of the remaining vital bacteria by live/dead staining, revealed the presence of significantly more vital cells in the CHX-treated initial biofilm (17.28%), compared to the initial biofilm treated with aPDT and THPTS (9.4%), whereas significantly more vital cells remained in the mature biofilm after aPDT treatment (25.67%) in comparison to mature biofilm treated with CHX (16.44%). This significant difference in the quantity of remaining vital cells in the CHX and THPTS treated mature oral biofilms, could be verified by CLSM images. Taking into consideration that the extracellular matrix of in situ biofilms contains a variety of polymeric substances, such as carbohydrates, nucleic acids, residues of bacterial components and extracellular enzymes and that each of these components is capable of interacting with THPTS, it is conceivable that the THPTS was partially neutralized in the mature biofilm and that its photoinactivation potential was blocked to a certain extent [22]. THPTS is a cationic substance and can, e.g., bind to the negatively-charged lipopolysaccharides (LPS) of Gram-negative bacteria, or to the peptidoglycan of Gram-positive bacteria, as has previously been described for the photosensitizer toluidine blue [23,24]. In addition, the THPTS concentration or the incubation time of the photosensitizer investigated in this study could have limited aPDT’s efficiency in mature biofilm. The situation is different in the initial biofilm because the pellicle is much thinner than that of the mature biofilm. The thin initial biofilm allows THPTS to eradicate most of the microorganisms. Neither the low THPTS concentration investigated nor the incubation time, can prevent the high eradication so that only 9.4% of the bacteria were still viable after aPDT. In contrast, the CHX treatment revealed similar vitality rates in the initial and mature biofilms (17.28% vs. 16.44%), which indicates that CHX’s efficiency is influenced by the same existing factors in both the initial and the oral biofilms. Presumably, saliva components have a neutralizing effect on CHX, as reported by an earlier study [25]. It should be borne in mind that CHX cannot be used over a long time period as it has some adverse effects, such as cell toxicity [26]. Furthermore, the possibility of oral bacteria being resistant to CHX, as well as cross-resistances to antibiotics, cannot be excluded if CHX is used intensively in the oral cavity [27].

Further studies should examine the efficiency of aPDT in relation to different concentrations and incubation times of the photosensitizer, THPTS. Furthermore, additional examinations should be performed to learn whether there are any changes in biofilm composition after aPDT treatment. Taking into consideration that as has recently been reported, Gram-negative bacteria are less susceptible to THPTS in aPDT than Gram-positive bacteria, a species level identification of the remaining oral microorganisms should be performed. This could clarify whether aPDT with THPTS has the potential to destroy the ecological balance, which is a precondition for biofilm formation and thus for biofilm associated infections.

## 5. Conclusions

In conclusion, antimicrobial photodynamic inactivation using VIS + wIRA in combination with THPTS has bactericidal effects on in situ-formed initial and mature oral biofilms and is therefore capable of eradicating oral bacteria within these niches. Because these bacteria cannot develop resistance to aPDT, this technique can be used to eradicate microorganisms which are resistant to CHX. Taking into consideration the healing effects of wIRA on human tissue, aPDT using VIS + wIRA in combination with THPTS is a promising technique for the treatment of peri-implantitis, periodontitis and endodontic infections. Further clinical studies are needed in the future to also evaluate this technique’s potential in combination with other photosensitizers to be used within the oral cavity.

## Data Availability

Data available on request due to restrictions eg privacy or ethical. The data presented in this study are available on request from the corresponding author.
